# Factors explaining priority setting at community mental health centres: a quantitative analysis of referral assessments

**DOI:** 10.1186/s12913-014-0620-3

**Published:** 2014-12-14

**Authors:** Sverre Grepperud, Per Arne Holman, Knut Reidar Wangen

**Affiliations:** Department of Health Management and Health Economics, University of Oslo, PO 1089, N-0317 Oslo, Norway; Lovisenberg Diakonale Hospital, N-0440 Oslo, Norway

**Keywords:** Access, Admissions, Criteria for prioritization, Specialized mental care, Referrals

## Abstract

**Background:**

Clinicians at Norwegian community mental health centres assess referrals from general practitioners and classify them into three priority groups (high priority, low priority, and refusal) according to need where need is defined by three prioritization criteria (severity, effect, and cost-effectiveness). In this study, we seek to operationalize the three criteria and analyze to what extent they have an effect on clinical-level priority setting after controlling for clinician characteristics and organisational factors.

**Methods:**

Twenty anonymous referrals were rated by 42 admission team members employed at 14 community mental health centres in the South-East Health Region of Norway. Intra-class correlation coefficients were calculated and logistic regressions were performed.

**Results:**

Variation in clinicians’ assessments of the three criteria was highest for effect and cost-effectiveness. An ordered logistic regression model showed that all three criteria for prioritization, three clinician characteristics (education, being a manager or not, and “guideline awareness”), and the centres themselves (fixed effects), explained priority decisions. The relative importance of the explanatory factors, however, depended on the priority decision studied. For the classification of all admitted patients into high- and low-priority groups, all clinician characteristics became insignificant. For the classification of patients, into those admitted and non-admitted, one criterion (effect) and “being a manager or not” became insignificant, while profession (“being a psychiatrist”) became significant.

**Conclusions:**

Our findings suggest that variation in priority decisions can be reduced by: (i) reducing the disagreement in clinicians’ assessments of cost-effectiveness and effect, and (ii) restricting priority decisions to clinicians with a similar background (education, being a manager or not, and “guideline awareness”).

## Background

The literature on prioritization in health care is mainly concerned with cost-effectiveness analyses and studies on priority-setting policies (macro- and meso-level) while priority setting at the micro-level (clinical level) is given less attention [1-7]. Priority setting at the clinical level is primarily a screening system (gatekeeping) for those seeking elective health care services and typical priority decisions made are: (i) admission or not, (ii) waiting time, (iii) length of treatment, and (iv) type of treatment (e.g. inpatient or outpatient). The same decisions are often supported by recommendations and instructions to meet social objectives such as: (i) treating those with different needs differently (vertical equity), and (ii) treating those with similar needs similarly (horizontal equity).

Quantitative studies on factors explaining priority setting are important for understanding to what extent social objectives are being fulfilled and for identifying effective policy measures [8]. Questions of interest are (i) do the prevailing criteria for prioritization actually play roles or not? (ii) If they do, how important are they? (iii) Do clinicians interpret the criteria similarly? (iv) Do non-clinical factors impact priority decisions? And (v) do organisational characteristics (clinical milieus, management, resource availability) matter? In this study a quantitative analysis of data from clinicians’ rating of referrals submitted to Norwegian community mental health centres (we refer to these as ‘centres’ in the following) is conducted. In a previous study, the same data were used to analyze the degree of inter-rater reliability with respect to priority setting [9].

Previous work on somatic health care services in relation to priority setting at the micro-level include studies on waiting times for general surgery [10,11], referral policies of physiotherapists [12,13] and qualitative studies on factors behind the institutional adoption of various technologies [14-17]. Literature on mental health care services that to some extent relates to micro-level prioritization issues includes studies on the assessment of severe mental illnesses and on the quality of referral letters and processes. Conclusions from this literature are: (i) professionals disagree on who constitutes the severely mentally ill [18-21], (ii) referral quality varies between general practitioners (GPs) and is often poor [22,23], (iii) in their referral letters, GPs underestimate the severity of symptoms [23], and, (iv) there is a primary vs. secondary care disagreement on referrals [24].

In Norway, prioritization (rationing) came on the political agenda in the mid-1980s because of the increase in the number of patients awaiting specialized treatment. In 1987, the government convened a commission to set forth criteria for prioritization [25-27] that proposed “severity of disease” as the only criterion. Ten years later, a second commission suggested three criteria [28]: (1) the patient has a condition with reduced prognosis related to life expectancy or quality of life if health care is delayed; (2) the patient has an expected effect of health care; and (3) there is a reasonable relation between costs and the effectiveness of the service (*p*, 646, [29]). We refer to these three criteria in the order presented above, as severity, effect and cost-effectiveness.

The second commission’s criteria were implemented in the Norwegian Patients’ Rights Act [30] with the intention to be used at all levels of the health care system (macro, meso, and micro) and in both sectors (somatic and mental health). Furthermore, all elective patients should be classified into one of three priority groups [31]: (i) no need for specialized treatment, (ii) in need of treatment, (iii) in need of necessary treatment (within an individual waiting time guarantee). In the following, we refer to the three priority groups respectively, as refusals, low priority and high priority.

Specialized mental health care in Norway is mainly supplied by psychiatric departments in general hospitals and centres. The psychiatric departments have wards (acute and other specialized inpatient wards) and are financed by fixed budgets, whereas the centres mainly provide outpatient services and have, to some degree, activity-based revenues. There are about 75 centres in Norway with an average catchment area of 65,000 adults and each centre is organized into departments in which there are several units [32]. GPs submit referral letters to their local centre, on the basis of which decisions to admit patients or not are made. The referral letters are not standardized [33]. The referral assessment process was organized differently across centres, at the time data were collected, with some having a single assessor, others having a joint admission team and still others having more than one team [9].

In recent years, various instructions, manuals and guidelines for the mental health care sector have been published to: (i) improve the organization of the referral assessment process, (ii) interpret the criteria of prioritization, and (iii) aid the centres in applying the same criteria for different diagnoses and conditions [34,35]. However, the prioritization process has not been supported by any validated instruments.

The specific aims of this study were to: (i) measure the degree of inter-rater reliability for the three prioritization criteria, (ii) study whether or not, and to what extent, priority setting was influenced by the same criteria, and (iii) investigate whether rater characteristics and factors at the organisational level (non-clinical factors) impact priority setting.

## Methods

### Study setting

This study was conducted at centres in the South-East Health Region of Norway during April and May 2009. Clinicians who took part in the assessment of referrals at the centres were independently asked to set priority on 20 anonymized referrals (case vignettes). The Regional Ethical Committee for Medical Research had no objections to this study because the referrals were fully anonymized.

### The test panel

Sixty-nine clinicians, all being involved with micro-level priority setting at 34 centres on a regular basis, were invited to participate. Forty-two clinicians at 16 centres agreed to participate, which was a response rate of 61%. Our study used data on individual ratings, and 14 of the 16 centres provided such data. The sample consisted of 840 individual ratings (42 clinicians and 20 referrals) but most variables had some missing values (confer Table 1).

**Table 1:** **Descriptive statistics**

**Variables**	**N**	**Mean (proportion)**	**Std. Dev.**	**Min**	**Max**
Priority group (the three-point version) Severity:	812	2.5	0.81	1	3
GAF-F	786	48.7	11.2	10	90
GAF-S	794	48.4	11.2	10	80
SumGAF	784	97.3	21.1	20	170
MinGAF	784	46.1	10.7	10	80
Profession					
Psychiatrist	840	(0.40)			
Psychologist	840	(0.36)			
Education	820	(0.88)			
Manager	820	(0.63)			
Rating experience	840	(0.67)			

(N = number of observations).

### Referrals, forms and variables

The 20 referrals used in this study were selected from a collection of 600 anonymized referrals submitted to five centres during 2008. The referrals reflected variation in symptoms, conditions (health state) and diagnosis (type of disorders), which made it likely that these patients would be rated into different priority groups. More details on the selection of referrals are available from a previously published study [9].

The form designed for this study was sent to the clinicians together with the 20 referrals. The clinicians were first asked to rate each referral according to effect, cost-effectiveness and severity (see the Background section for further details), as described below. Thereafter, the clinicians were asked to rate each referral into the priority groups defined by the national prioritization guidelines (high priority, low priority and refusal). Finally, the clinicians were asked to answer some background questions.

Four versions of the dependent variable were used for the priority group. The first version (model I) used a three-point scale where refusals =1, low priority =2 and high priority =3. For the remaining three versions (two-point scales), the coding was as follows: refusals =1 and low and high priority =0 (model II); low priority =0 and high priority =1 (model III); and, refusals =0 and low priority =1 (model IV).

Observations with missing values were omitted so that the sample size for Models I and II was 724. In models III and IV observations were omitted based on the dependent variable (model III: refusals; model IV: high priority) yielding sample sizes of 592 and 217, respectively. In Models II and III additional observations (52 and 14, respectively) were excluded from analysis because some of the centre specific constant terms were perfectly correlated with the dependent variable.

Both effect and cost-effectiveness were measured by using four-point Likert scales ranging from 1 to 4. For effect, the value 1 referred to “no expected effect” while 4 referred to “a significant expected effect”. For cost-effectiveness, the value 1 referred to “a very low relation between costs and the effectiveness of the service” while 4 referred to “a high relation between costs and the effectiveness of the service.”

To measure severity, we applied the Global Assessment Scale (GAF). There were three reasons for this. First, GAF is a generic scoring system constructed as an overall (global) measure of how patients are doing and rates psychological, social and occupational functioning [36]. Second, GAF has received much attention in the research literature [37-44]. Third, GAF is well known to Norwegian clinicians because it is used in routine clinical practice [42,45]. Each referral was rated according to the dual (split version) of GAF, which provides separate scores for symptoms (GAF-S) and functioning (GAF-F), with 100 scoring possibilities each and where lower values represent more severe cases. Based on the scores for GAF-F and GAF-S, we constructed two additional variables. The first, SumGAF, was calculated by taking the sum of the two scores. The second, MinGAF, was calculated by taking the minimum value, which was the more severe of the two GAF-scores.

Respondents reported their profession (psychiatrist, psychologist or other), education (specialist or not), being a manager or not and rater experience (years). In addition, they were asked to answer three questions concerned with knowledge, experience and training in the use of priority setting and guidelines. The variables of education (specialist =1, non-specialist =0), manager (yes =1, no =0), rater experience (more than two years =1; two years or less =0), psychiatrist (psychiatrist =1 and psychologist or other =0), and psychologist (psychologist =1 and psychiatrist or other =0), were coded as dummy variables.

An index variable was designed to measure the degree of awareness about the guidelines for priority setting. This variable (guideline awareness) was constructed by adding the answers to the following three questions: (i) are you well informed about the Act of Patients’ Rights? (yes =1, no =0); (ii) in the last year, have you applied the guidelines for priority setting in mental health care? (yes =1, no =0); and (iii) have you received any training in applying the guidelines for priority setting in mental health care (yes =1, no =0)?

### Statistical analysis

First, descriptive statistics were calculated. The degree of agreement across raters on each of the three criteria of prioritization was measured using intra-class correlation (ICC) analysis. A two-way random effect model was applied, ICC (2,1), where a random sample of *k* judges (raters) were selected from a larger population, and each judge (rater) rated *n* targets (referrals) [46]. Missing ratings caused a reduction in the number of observations. To correct for these losses, missing observations were replaced with mean values. Logistic regression analyses (ordered and binary) were applied to identify explanatory variables that impacted priority setting. Since our data set (individual ratings) exhibits a hierarchical structure in which clinicians belong to 14 different centres, logistic models with centre-specific constant terms (fixed effects) were applied.

For the purpose of interpretation suggested labels that we may use for ICC are [47]: (1) ICC < 0.20 (slight agreement); (2) 0.21–0.40 (fair agreement); (3) 0.41–0.60 (moderate agreement); (4) 0.61–0.80 (substantial agreement); (5) >0.80 (almost perfect agreement).

## Results

Table 1 presents the variables in terms of number of observations, means or proportions, standard deviations and range. The standard deviations for the three GAF variables that range from 1 to 100 were more or less similar. For the priority group variable, 547 (67.3%) were rated as having high priority, 99 (12.1%) were rated as having low priority, while 166 (20.4%) were not given any priority (refusals). Five of the centres each had 4.8% of the total ratings, four had 7.1% each, while the remaining five had 9.5% each. For the effect variable, the shares that responded values 1 to 4 were: 2%, 18%, 72% and 8% (*N* =821). For cost-effectiveness the shares were: 5%, 15%, 53% and 28% (*N* =820). Forty percent of the raters were psychiatrists, 36% were psychologists, 88% were specialists, 63% were acting as managers (most of them as unit managers) and 67% had a priority rating experience of two years or more. For the guideline awareness index, the shares that responded values 0 to 3 were: 5%, 12%, 55% and 29% (*N* =840). More detailed information on the background variables of the participating raters was published previously [9].

Table 2 shows that the single-measure ICCs (two-way random model, absolute agreement) for the priority group, effect, cost-effectiveness and three of the severity variables (GAF-S, GAF-F, SumGAF) varied considerably (from 0.29 to 0.67). The ICC for the three GAF variables (from 0.55 to 0.67) was higher than those for the priority group (0.43), effect (0.34), and cost-effectiveness (0.29). The three GAF variables did not differ significantly; however, both SumGAF and GAF-S variables were significantly higher than effect and cost-effectiveness (5%).

**Table 2:** **The level of agreement measured by intra-class correlation coefficients (ICCs)**

		**Single-measure ICCs (replaced missing observations)**
Priority group^a^	(1–3 scale)	0.43 (0.30–0.62)
SumGAF	(0–200 scale)	0.67 (0.53–0.81)
GAF-S	(0–100 scale)	0.64 (0.52–0.77)
GAF-F	(0–100 scale)	0.55 (0.43–0.70)
Effect	(1–4 scale)	0.34 (0.22–0.53)
Cost-effectiveness	(1–4 scale)	0.29 (0.19–0.48)

A two-way random model (2.1) absolute agreement, between 42 individual raters over 20 referrals. Confidence interval: 95%-level. N =840 (replacement of missing observations).

^a^These numbers are previously reported in [9].

Logistic regression analyses were conducted (see Table 3). First, an ordered logistic regression was performed to identify factors producing ratings in higher priority groups (model I). The next three regressions were binary logistic regressions. In model II, all ratings were classified as admitted (high and low priority) or non-admitted. Models III and IV excluded some ratings and can be regarded as conditional models; model III distinguished between those given a high priority and those given a low priority; acting as a benchmark, model IV distinguished between those given a low priority and those who were non-admitted.

**Table 3:** **Multivariate logistic regressions with fixed effects: factors affecting priority setting**

	**Ordered logistic regression**	**Binary logistic regressions**		
**Determinants**	**Model I (n =724)**	**Model II (n =672) High and low priority vs. refusals**	**Model III (n =578) High priority vs. low priority**	**Model IV (n =217) Low priority vs. refusals**
Severity (MinGAF)	−0.11 (0.01), *p* =0.00	−0.09 (0.02), *p* =0.00	−0.12 (0.02), *p* =0.00	−0.03 (0.02), *p* =0.14
Effect	0.95 (0.27), *p* =0.00	0.36 (0.35), *p* =0.30	1.91 (0.43), *p* =0.00	−0.37 (0.38), *p* =0.33
Cost-effectiveness	1.64 (0.23), *p* =0.00	1.89 (0.30), *p* =0.00	1.27 (0.37), *p* =0.00	1.15 (0.32), *p* =0.00
Profession: Psychiatrist (=1) or not (=0)	0.15 (0.33), *p* =0.65	0.34 (0.45), *p* =0.45	0.23 (0.47), *p* =0.63	0.28 (0.56), *p* =0.61
Profession: Psychologist (=1) or not (=0)	0.36 (0.36), *p* =0.32	0.93 (0.50), *p* =0.06	−0.21 (0.52), *p* =0.69	0.95 (0.57), *p* =0.09
Education: Specialist (=1) or not (=0)	−1.19 (0.47), *p* =0.01	−1.75 (0.63), *p* =0.01	−0.25 (0.64), *p* =0.70	−1.49 (0.80), *p* =0.06
Manager (=1) or not (=0)	−0.61 (0.30), *p* =0.04	−0.38 (0.39), *p* =0.34	−0.45 (0.45), *p* =0.32	−0.22 (0.47), *p* =0.64
Rating exp. (=1 if 2 years) (=0 if <2 years)	0.13 (0.25), *p* =0.61	0.39 (0.33), *p* =0.24	−0.17 (0.35), *p* =0.64	0.39 (0.39), *p* =0.33
Guideline awareness	−0.29 (0.17), *p* =0.09	−0.36 (0.22), *p* =0.10	−0.20 (0.26), *p* =0.44	−0.17 (0.28), *p* =0.54
Fixed effects (centres)				
A	0.35 (0.60), *p* =0.56	−0.20 (0.77), *p* =0.79	0.97 (0.87), *p* =0.27	−0.49 (0.95), *p* =0.60
B	1.20 (0.67), *p* =0.07	0.66 (0.85), *p* =0.43	1.34 (0.99), *p* =0.18	0.04 (0.95), *p* =0.97
C	1.35 (0.58), *p* =0.02	1.08 (0.75), *p* =0.15	1.32 (0.85), *p* =0.12	0.70 (0.86), *p* =0.42
D	1.89 (0.74), *p* =0.01	1.47 (1.04), *p* =0.16	2.11 (0.99), *p* =0.03	0.42 (1.32), *p* =0.75
E	0.22 (0.64), *p* =0.73	−0.07 (0.79), *p* =0.93	0.91 (0.95), *p* =0.34	−0.57 (1.01), *p* =0.57
F	1.68 (0.64), *p* =0.01	–^a^	0.78 (0.79), *p* =0.33	–^a^
G	1.07 (0.73), *p* =0.14	1.09 (1.06), *p* =0.31	0.97 (0.97), *p* =0.32	0.65 (1.19), *p* =0.59
H	0.75 (0.54), *p* =0.16	0.92 (0.68), *p* =0.18	0.48 (0.78), *p* =0.53	0.62 (0.76), *p* =0.41
I	0.58 (0.63), *p* =0.36	0.28 (0.86), *p* =0.75	1.90 (0.84), *p* =0.28	0.03 (1.06), *p* =0.98
J	0.42 (0.54), *p* =0.44	−0.08 (0.67), *p* =0.91	1.03 0.82), *p* =0.21	−0.68 (0.83), *p* =0.41
K	0.30 (0.51), *p* =0.56	−0.05 (0.63), *p* =0.94	0.96 (0.79), *p* =0.23	−0.35 (0.76), *p* =0.65
L	0.19 (0.83), *p* =0.82	−0.93 (0.94), *p* =0.33	–^a^	–^a^
M	2.55 (0.78), *p* =0.00	2.87 (1.05), *p* =0.01	1.85 (1.14), *p* =0.11	2.017 (1.17), *p* =0.07
Constant	–	0.88 (1.32), *p* =0.51	−1.80 (1.60), *p* =0.26	0.17 (1.54), *p* =0.91
Pseudo R^2^	**0.36**	**0.41**	**0.36**	**0.14**

Coefficients, (standard errors) and p-values. (n =217–724).

^a^These centres are ignored since all scores for these centres belong to one priority group only (to avoid dummy traps).

We ran regressions with each of the four GAF variables as measures of severity. All four variables produced more or less similar results as concerns estimated coefficients and significance levels for all independent variables. We chose to report results for the MinGAF variable in the regressions, which are presented in Table 3. This table shows that severity was strongly significant in models I, II and III, Cost-effectiveness was strongly significant in all four models, whereas effect was significant only in models I and III.

For model I, we observed that three of the rater characteristics were significant: guideline awareness (9% level), education (1% level) and manager (4% level). Consequently, the probability of being assigned to a higher priority group increased with a higher severity, a higher effect, higher cost-effectiveness, if the rater was a non-specialist, a non-manager, or if the rater had low guideline awareness. Comparing the magnitude of effect and cost-effectiveness, both measured on a four-point scale, the latter had the strongest impact on priority setting. It was also observed that the magnitude from effect was weaker in model I compared with model III.

Comparing model II (the decision to rate patients into admitted and non-admitted) with model III (the decision to rate admitted patients into either high priority or low priority), we observed that effect is insignificant in model II and strongly significant in model III. In addition, all rater characteristics were insignificant in model III while three were significant in model II. Other findings of interest include: (i) across all four models, centre M differed from the other centres, (ii) about one third of the fixed effects of model I were significant (7% level) and (iii) the profession dummy variables confirm that psychologists gave higher priority than the other professional groups; however, this effect was significant only in model II (6%).

## Discussion

The main findings of this study were that: (i) all three criteria of prioritization had strongly significant coefficients, (ii) non-clinical factors (centre and rater characteristics) explained variation in priority decisions and (iii) the importance of some variables changed across priority decisions.

In our study, GAF-scores were used to measure severity. All regression analyses performed confirmed that all four GAF variables have important and significant effects on priority setting. In Norway, clinicians typically apply GAF to score patients at the first and last treatment session (routine clinical practice). In addition, clinicians are also invited to practice on the use of GAF (staff training) by rating a set of case vignettes that become available to them on demand. Such calibration exercises are found to reduce GAF score variation across clinicians [43,44]. At present, guidelines and recommendations for the mental health care sector do not mention GAF as an instrument that can aid admission teams. A natural question now becomes whether the raters’ perception of severity, as defined in the national guidelines of prioritization, can be meaningfully “translated” into GAF scores. This is a question that should be addressed in future research.

The significant non-clinical factors were education, manager status, profession and guideline awareness. These findings suggest that if priority setting were left to more homogenous raters, the degree of agreement would be improved. An additional non-clinical factor was captured via some of the fixed effects. Other factors being equal, centre M gave a higher priority to all patients compared with other centres and 1/3 of the fixed effects of model I became significant. These findings say that there are effects at the unit (organisational) level, however we do not know what particular factors that play a role. Possible candidates are variations in clinical practice, organization and resource availability. We know from a previous study that resource availability (budget relative to health risks) varies significantly across Norwegian centres [48]. Given that resource availability plays such a role, variation in priority decisions can be reduced by reallocating resources (budgets) to achieve a balanced capacity across community mental health centres. The importance of non-clinical factors (rater characteristics and institutions) is a finding identified by other studies on rating behaviour as well [40,41].

The observation that some variables changed in importance across priority decisions is best illustrated by comparing model II and III. In model III, the priority decision (patients admitted into high or low priority groups) was influenced by all three criteria and none of the rater characteristics, whereas for model II the priority decision (admitted vs. non-admitted) was unaffected by one criterion (effect) at the same time that three rater characteristics were significant. These findings could point to structural differences between the two decisions: One possible explanation is budgetary (resource) constraints. The priority decision of model II determined the actual number of patients to be given treatment (admitted patients) which has a direct bearing on the need for resources whereas the priority decision of model III only concerns those already admitted. Accepting this explanation, we may conclude that: (i) raters give more weight to cost-effectiveness and less weight to effect for priority decisions with budgetary implications, and (ii) being a specialist, a non-psychologist, and having high guideline awareness reduces the probability of being classified into the highest priority group for the priority decision with budgetary implications, only.

Our findings from the ICC analyses confirm variation in raters’ assessments of all three prioritization criteria; however, the degree of agreement is significantly higher for severity (for all GAF variables) than for effect and cost-effectiveness. Former studies on inter-rater reliability and GAF ratings have found that: (i) intra-centre reliability is higher than inter-centre reliability [38], (ii) reliability increases with clinical experience [45], and (iii) inter-rater liability is moderate [49,50] or satisfactory [43,44]. Compared with former studies, inter-rater reliability for the GAF ratings in our study were only moderate; despite this, they were higher than the inter-rater reliability for both effect and cost-effectiveness. There are several explanations for these findings. First, GAF instruments are well known to our respondents since they have been used in routine clinical practice for decades. Second, until about a decade ago, disease severity was the only prioritization criterion whereas effect and cost-effectiveness are recently introduced criteria implying that clinicians have less experience with assessing such dimensions. Third, unlike severity, effect and cost-effectiveness involve predictions about future outcomes and former studies have confirmed that clinicians are very poor at making predictions on the basis of referral letters [51].

The ICCs suggest that effect and cost-effectiveness are the most important contributors to low inter-rater reliability with respect to priority groups. This conclusion, however, rests upon the assumption that all three criteria were given similar weights by the raters as a group. The estimated coefficients of the ordered logistic regression only confirm that all three criteria were given “some” weight and that a relative comparison was difficult because of different measurement scales. However, what we did observe was that the weighting changed across the priority decisions. This was particularly so for effect because it was insignificant and weak in model II while strong and significant in models I and III. Therefore, a reduction in the variability of the raters’ assessments of effect would not improve the degree of agreement when it comes to priority setting between admitted and non-admitted patients. It should be noted that the main goal is to be in line with the intention of the guidelines for priority setting and not reducing variation as such.

Many studies have found that the quality of referral letters was relatively low [22,23,33]. Such findings suggest that some type of standardization might produce more precise and structured referrals that again would improve the prioritization processes. Whether standardization will actually improve such processes or not should be an area of future investigations. There are additional policy measures that might work, such as improving clinicians’ awareness and understanding of prioritization, operationalization of the prioritization criteria and the development of instruments that may aid raters in assessing the same criteria.

Our study has some potential limitations. First, the participating centres may differ systematically from those that did not participate, which creates a selection bias. Second, the rating of referrals was a hypothetical exercise that may produce results different from actual priority choices. Third, we studied individual ratings, while referrals in practice are addressed by admission teams at about 50% of the centres [9]. Fourth, the index variable (guideline awareness) follows from asking three questions, each with only two response categories (yes or no), thus it becomes a question as to whether this variable becomes too simple to capture the degree of awareness about the guidelines for priority setting.

## Conclusions

The main findings of this study were that (i) clinicians disagree on the three criteria for prioritization, (ii) this disagreement is strong for effect and cost-effectiveness, but is weaker for severity, (iii) the weight varies across criteria and across the priority decision studied, and (iv) non-clinical factors (rater characteristics and inter-centre differences) impact priority decisions. In sum, these findings point to the: (i) complexity of the prioritization processes, especially when there are several criteria, and, (ii) challenges associated with reaching social objectives such as vertical and horizontal equity. Our findings suggest the presence of a policy trade-off; limiting the number of criteria (e.g. by using severity only) might improve horizontal equity. However, this will occur at the expense of vertical equity because then priority would be given to groups with lesser needs as defined by the national priority guidelines.

Our empirical results identified measures that may reduce the variation in priority setting across clinicians such as: (i) improving inter-rater reliability for effect and cost-effectiveness, and (ii) leaving priority setting to raters with a similar background. In addition, our findings point to some promising candidates toward improving inter-rater reliability, such as a better referral quality, the operationalization of criteria, and an improved awareness of the prioritization process. More research on the costs and benefits of such measures is in demand.
